# The role of genotype in Familial Mediterranean Fever

**DOI:** 10.1186/1546-0096-12-S1-P261

**Published:** 2014-09-17

**Authors:** Semanur Özdel, Zeynep Birsin Özçakar, Seda Sahin, Mesiha Ekim, Atilla Elhan, Fatos Yalcinkaya

**Affiliations:** 1 Pediatric Rheumatology, Ankara University, Ankara, Turkey; 2 Pediatrics, Ankara University, Ankara, Turkey; 3 Biostatistics, Ankara University, Ankara, Turkey

## Introduction

Familial Mediterranean Fever (FMF) is an autosomal recessive disease, characterised by recurrent, self limited attacks of fever with serositis. The gene responsible for FMF, designated as *MEFV*, encodes pyrin.

## Objectives

The aim of this study was to compare the demographic and clinical features of FMF patients with heterozygous *MEFV* mutations to those with homozygous or compound heterozygous mutations.

## Methods

Files of patients who had been seen in our department (during routine follow-up visits) between January 2013 and January 2014 were retrospectively evaluated. Six predominant mutations (p.M694V, p.M680I, p.M694I, p.V726A, p.K695R, p.E148Q) in the *MEFV* gene were studied in our center. Patients were divided into two groups: group I included patients with heterozygous mutations and group II included patients with homozygous or compound heterozygous mutations.

## Results

The study group comprised 263 FMF patients (145 females, 118 males) with a mean age of 9.7 ±5.2 years. There were 83 patients in group I and 180 patients in Group II. Although age at disease onset and clinical findings did not differ between the two groups, age at onset of colchicine therapy was lower in group II (p<0,05) patients. Family history of FMF was more frequently detected in group II (p=0,016). Acute phase reactant levels during the attacks before colchicine therapy and the attack-free period after colchicine therapy were higher in group II (p<0,05). Median PRAS severity score and final colchicine dosages were also higher in group II (p<0,05) patients.

## Conclusion

As an expected finding FMF patients with homozygous and compound heterozygous mutations have more severe disease during childhood period.

## Disclosure of interest

None declared.

